# Investigation into Improving the Aqueous Solubility of the Thieno[2,3-*b*]pyridine Anti-Proliferative Agents

**DOI:** 10.3390/molecules23010145

**Published:** 2018-01-11

**Authors:** Ayesha Zafar, Lisa I. Pilkington, Natalie A. Haverkate, Michelle van Rensburg, Euphemia Leung, Sisira Kumara, William A. Denny, David Barker, Ali Alsuraifi, Clare Hoskins, Jóhannes Reynisson

**Affiliations:** 1 School of Chemical Sciences, University of Auckland, 23 Symonds Street, 1142 Auckland, New Zealand; ash_imran@hotmail.com (A.Z.); lisa.pilkington@auckland.ac.nz (L.I.P.); nhav422@aucklanduni.ac.nz (N.A.H.); m.vanrensburg@auckland.ac.nz (M.v.R.); d.barker@auckland.ac.nz (D.B.); 2 Auckland Cancer Society Research Centre and Department of Molecular Medicine and Pathology, University of Auckland, 1142 Auckland, New Zealand; e.leung@auckland.ac.nz (E.L.); s.kumara@auckland.ac.nz (S.K.); b.denny@auckland.ac.nz (W.A.D.); 3 Institute for Science and Technology in Medicine, Keele University, Stoke-on-Trent ST4 7QB, UK; a.t.y.alsuraifi@keele.ac.uk (A.A.); c.hoskins@keele.ac.uk (C.H.)

**Keywords:** morpholine substitution, 1*H*-pyrrolo[2,3-*b*]pyridine, molecular modelling, nano aggregates, polymer formulation

## Abstract

It is now established that the thieno[2,3-*b*]pyridines are a potent class of antiproliferatives. One of the main issues encountered for their clinical application is their low water solubility. In order to improve this, two strategies were pursued. First, a morpholine moiety was tethered to the molecular scaffold by substituting the sulphur atom with nitrogen, resulting in a 1*H*-pyrrolo[2,3-*b*]pyridine core structure. The water solubility was increased by three orders of magnitude, from 1.2 µg/mL (**1**-thieno[2,3-*b*]pyridine) to 1.3 mg/mL (**3**-pyrrolo[2,3-*b*]pyridine), however, it was only marginally active against cancer cells. The second strategy involved loading a very potent thieno[2,3-*b*]pyridine derivative (**2**) into a cholesteryl-poly(allylamine) polymer matrix for water solubilisation. Suppression of human pancreatic adenocarcinoma (BxPC-3) viability was observed to an IC_50_ value of 0.5 μg/mL (1.30 μM) in conjunction with the polymer, which is a five-fold (×5) increase in potency as compared to the free drug alone, demonstrating the utility of this formulation approach.

## 1. Introduction

**Figure 1:**
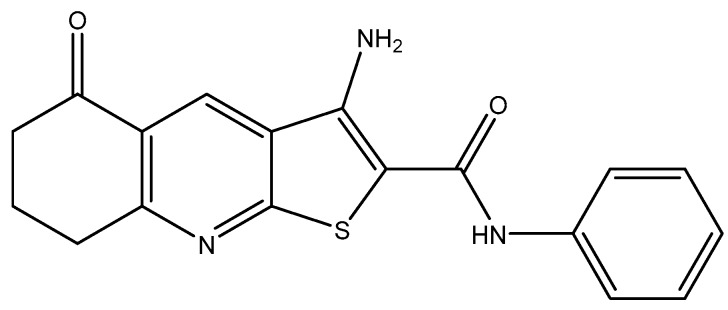
The structure of the anticancer thieno[2,3-*b*]pyridine **1** (3-amino-5-oxo-*N*-phenyl-5,6,7,8-tetrahydrothieno[2,3-*b*]quinoline-2-carboxamide).

The class of thieno[2,3-*b*]pyridines are potent anti-proliferative agents against a number of tumour cell lines [1,2,3] and their basic molecular structure is shown in Figure 1. The efficacy of the thieno[2,3-*b*]pyridines was discovered by virtual high throughput screen (vHTS) against the phospholipase C-γ2 (PLC-γ2) isoform, a promising anticancer target [4]. The administration of thieno[2,3-*b*]pyridines causes the breast cancer cell line MDA-MB-231 to be severely growth-restricted, rounding and blebbing of the plasma membrane, G_2_/M phase population to increase in the cell cycle, and a decrease in motility [5,6]. These effects on MDA-MB-231 are more in line with the inhibition of the PLC-δ1 and δ2 isoforms, making them the most plausible target for this class of compounds [6]. However, it has been shown that other bio-molecular targets are modulated, contributing to the overall efficacy of the thieno[2,3-*b*]pyridines: tyrosyl-DNA phosphodiesterase I (TDP1) [7], the A_2A_ receptor (A_2A_AR) a G-protein coupled receptor (GPCR)[8], copper trafficking protein Atox [9], and tubulin [10,11].

A major obstacle in developing the thieno[2,3-*b*]pyridines into effective anticancer drugs is their lack of aqueous solubility. In order to derive a meaningful mouse pharmacokinetic profile the compound was required to be solubilised using a cyclodextrin (HP-β CD), a well-known solubilising method [6]. Furthermore, in a mouse xenograft study on the effect of thieno[2,3-*b*]pyridine administration, water solubility was the dose-limiting factor [7]. It is estimated that ~40% of drug discovery programmes are discontinued due to limited water solubility and associated poor pharmacokinetics of the lead compounds [12,13]. It is, therefore, evident that the water solubility of thieno[2,3-*b*]pyridines needs to be improved for meaningful clinical application. 

The aim of this study was to investigate two strategies to improve the water solubility of the thieno[2,3-*b*]pyridines. First, by adding a solubilising group to the molecular scaffold and, second, to load an active derivative in a formulation polymer.

## 2. Results and Discussion

**Figure 2:**
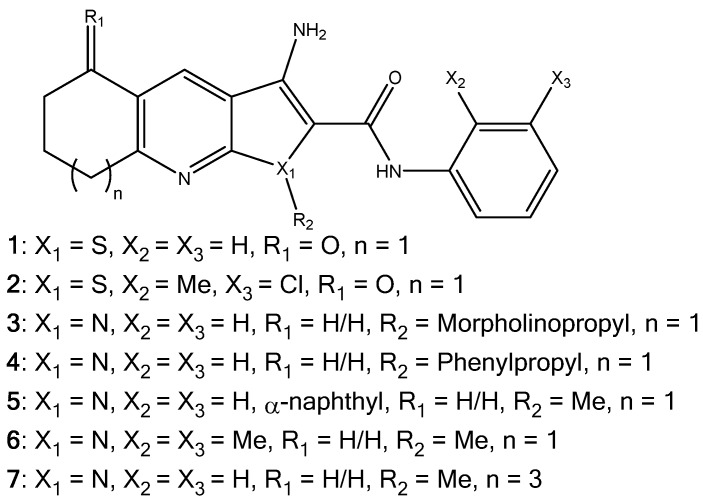
The molecular structures of the thieno[2,3-*b*]pyridines (**1**, **2**) and 1*H*-pyrrolo[2,3-*b*]pyridines (**3**–**7**).

Seven compounds were chosen for investigation into the improved aqueous solubility of the thieno[2,3-*b*]pyridines. Compounds **1** and **2** were selected due to their established anticancer profiles [1,2]. In order to attach a solubilising group to the thieno[2,3-*b*]pyridine scaffold, the sulphur atom was replaced with nitrogen creating the 1*H*-pyrrolo[2,3-*b*]pyridine molecular frame as seen for derivatives **3**–**7** in Figure 2. It has been previously shown that substitution of the sulphur atom for oxygen gave derivatives with comparable activity [3]. The standard solubilising group morpholine was attached resulting in derivative **3**, as a direct comparison a comparable phenyl derivative (**4**) was synthesised, and derivatives **5**–**7** had a methyl substitution on R_2_, but with different ring sizes and substitutions to gauge the effect of substituting sulphur with a nitrogen atom, in general. 

### 2.1. Synthesis of Pyrrolo[2,3-b]pyridine Derivatives

**Scheme 1:**
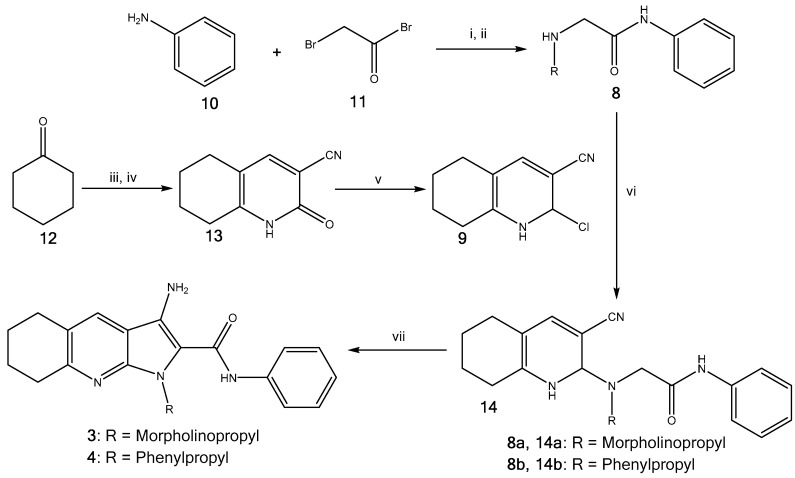
Synthesis of **3** and **4**. (i) Et_3_N (1.1 equiv.), CH_2_Cl_2_, r.t., 24 h, 88%; (ii) RNH_2_, EtOH, r.t., 24 h, **8a**,**b** 68%-quant.; (iii) Na (1 equiv.), MeOH (5.6 equiv.), ethyl formate (1 equiv.), r.t., 24 h, 42%; (iv) cyanoacetamide (1 equiv.), piperidinium acetate, H_2_O, reflux, 24 h, 29%; (v) POCl_3_ (1.3 equiv.), reflux, 24 h, 49%; (vi) KF (2.4 equiv.), DMSO, 120 °C, 24 h, **14a**,**b** 10–51%; (vii) KO*^t^*Bu (1.2 equiv.), THF, reflux, 30 min, **3** and **4** 90–100%.

Derivatives **1** and **2** were synthesised using previouslydescribed methods [3]. Furthermore, the pyrrolopyridines (**3**–**7**) were synthesised as described by Pilkington et al. [14]. As an example, pyrrolopyridines **3** and **4** were made by coupling amines **8a**,**b** with carbonitrile **9** (Scheme 1). Initially, aniline **10** was converted to bromophenylacetamide using bromoacetyl bromide **11** in the presence of triethylamine, which was then reacted with various amines to give *N*-substituted phenylacetamides **8a**,**b**. Carbonitrile **9** was prepared from cyclohexanone **12** in three steps; firstly, a methylenehydroxy salt was prepared by adding ethyl formate and cyclohexanone **12** to a solution of freshly prepared sodium methoxide. The pyridone was formed through addition of cyanoacetamide to the prepared salt, giving **13**, which was followed by chlorination using POCl_3_, providing carbonitrile **9**. *N*-substituted phenylacetamides **8a**,**b** were coupled with carbonitrile **9** in DMSO using KF to give nitriles **14a**,**b**, which then underwent cyclisation using KO*^t^*Bu to give the desired analogues **3** and **4** in excellent yields (90–100%).

### 2.2. Water Solubility Testing

The aqueous thermodynamic solubility [15] of derivatives **1**–**7** were measured using a standard HPLC (High Performance Liquid Chromatography) procedure [16]. Solubility is defined as the amount of substance that passes into solution in order to establish the equilibrium at constant temperature and pressure and, thus, produce a saturated solution. The water solubility (log S) and log P were calculated for comparison. The results are given in Table 1.

According to the experimental results, it is clear that compounds **1** and **2** are relatively insoluble with only single-digit µg/mL. This modest solubility can explain the low plasma concentration in mice of thieno[2,3-*b*]pyridines, i.e., only ~90 nM maximum concentration (C_max_) was observed after 10 mg/Kg intra-peritoneal injection [6].

Compound **3**, with a tethered morpholine moiety resulted in a solubility increase by three orders of a magnitude giving ~1.3 mg/mL as compared to **1**. As expected, substituting the morpholine moiety for a phenyl ring led to the lower solubility of derivative **4**. Interestingly, compounds **5**–**7** also have very poor solubility even though they were only substituted with a methyl group. 

The calculated solubilities follow the trend of their measured counterparts and for derivative **3** a reasonable correlation is seen (18.6%). For compounds **1**, **2**, **4**, **5**, **6**, and **7** the predicted values are too large by approximately one order of magnitude.

Interestingly, the log P values do not follow the solubility trends well except for derivative **4** with a high log P at 5.3, which is outside of drug-like chemical space. The other mainstream descriptors: molecular weight (MW), hydrogen bond donors (HD), hydrogen bond acceptors (HA) polar surface area (PSA) and rotatable bonds (RB) (see Table S1 in the Supplementary Materials) are all within the drug-like chemical space. It has been previously noted that the currently-used molecular descriptors like log P and log S do not entirely capture the required water solubility profiles of successful drugs [17].

### 2.3. Cell Proliferation

Thymidine uptake was used for viability testing on the MDA-MB-231 (breast) and HCT116 (colon) human tumour cell lines. This measurement reflects the anti-proliferative/cytotoxic effects of the compounds. A concentration of 1 μM was used. Very little activity was observed for the 1*H*-pyrrolo[2,3-*b*]pyridine derivatives **3**–**7** (see Table S2 in the Supplementary Materials). In comparison, derivative **2** has a measured IC_50_ of 163.5 nM against the MDA-MB-231 cell line using the thymidine uptake assay [6]. To corroborate these findings, derivatives **3**, **4**, and **6** were tested against the National Cancer Institute’s human tumour cell line panel (NCI60) [18]. Again, very modest or no activity was found (see NCI data in the Supplementary Materials). Derivative **6** was the most potent with 77% overall relative growth for the 60 cell lines as compared untreated cells (100% growth). The leukaemia cell lines K-562 (45.5% growth) and SR (47.7% growth) were the most affected followed by the melanoma MDA-MB-435 (53.9% growth) cell line. This is an interesting observation since the thieno[2,3-*b*]pyridines are often active against leukaemia cells and the MDA-MB-435 cell line, in particular [2,7,19,20]. It can, therefore, be concluded that the 1*H*-pyrrolo[2,3-*b*]pyridines are nearly inactive against cancer when compared to their thieno[2,3-*b*]pyridine counterparts. Again, this is in stark contrast to the thieno[2,3-*b*]pyridines, in particular for derivative **2**, with reported GI_50_ = 141 nM for MDA-MB-231 and GI_50_ = 67 nM for HCT116 [1,2,6].

### 2.4. Molecular Modelling

Compounds **1**–**7** were docked into the binding pockets of the PLC-δ1 [6], TDP1 [7], A_2A_AR [8], Atox [9], and tubulin-colchicine site [10,11], the reported biological targets for the thieno[2,3-*b*]pyridines. Reasonable scores were observed for the four scoring functions used for all of the targets, which are given in Tables S3–S7 in the Supplementary Materials. Based on the scores it may be deduced that derivatives **1**–**7** all interact with these targets, i.e., no obvious reduction in scores was seen.

**Figure 3:**
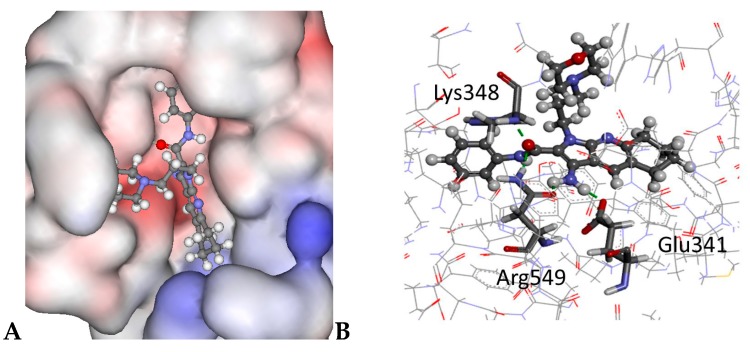
The docked configuration of **3**: (**A**) to the binding site of PLC-δ1 using the ChemPLP scoring function. The morpholine occupies a cleft on the protein surface, but has access to the water environment. The protein surface is rendered. Red depicts a positive partial charge on the surface, blue depicts negative partial charge and grey shows neutral/lipophilic areas; (**B**) Hydrogen bonds are depicted as green lines between the ligand and the amino acid residues Lys348, Arg549, and Glu341.

For PLC the thieno[2,3-*b*]pyridines and 1*H*-pyrrolo[2,3-*b*]pyridine scaffolds are predicted to fit in a similar fashion into the binding pocket (see Table S3 in the Supplementary Materials) and these conformations are in line with previous work [2,5]. The morpholine and phenyl side chains on derivatives **3** and **4** occupy a cleft on the protein surface as shown for **3** in Figure 3. Importantly, the morpholine moiety is exposed to the aqueous phase.

For TDP1 it is proposed that both histidine amino acid residues (His263 and His493) play a role in its biological function and the binding pocket was defined there [21]. Upon docking, compounds showed varied hydrogen bonding patterns (Table S4 in the Supplementary Materials), but a similar fit was predicted for all of them.

A common binding motif is reported for Atox involving polar interactions with side chains of arginine (Arg21), lysine (Lys60), and glutamic acid (Glu17) in order to block protein-protein interface for copper delivery [9]. Similar binding poses are predicted for **1**–**7**, e.g., for **2** the phenyl ring has a lipophilic contact with arginine (Arg21) and a π-π stacking with lysine (Lys60) is possible (see Table S5).

The A_2A_RA has reported binding motifs of stacking between aromatic moieties of the ligands and the Phe168 side chain of the receptor, as well as polar interactions with Asn253 and Glu169 side chains [8]. These interactions were predicted for compounds **1**–**7** and similar binding configurations were seen for the derivatives (see Table S6 in Supplementary Materials). Finally, the tubulin-colchicine site was investigated and **1**–**7** displayed similar stacking and H-bonding interaction as colchicine (see Table S7).

In all of the bio-molecular targets investigated, the chemical modifications made to the thieno[2,3-*b*]pyridine scaffold can be accommodated by the binding sites. These results suggest that other factors reduce the potency of the 1*H*-pyrrolo[2,3-*b*]pyridine derivatives **3**–**7** than binding to the established targets. This might include the very high solubility of **3**, preventing it passing into cell membranes, thus reducing its permeation. The very low solubility of analogues **4**–**7** prevents concentration build up in the cancer cells presenting a plausible explanation for their inactivity. In both scenarios offered the ligands do not reach the targets in high enough concentration to adversely affect the cancer cells. An alternative explanation is that the thieno[2,3-*b*]pyridines exert their anticancer efficacy by modulating an unknown molecular target. 

### 2.5. Polymer Encapsulation

A common strategy to facilitate water solubility for bioactive compounds is their formulation in a solubilising amphiphilic polymer (see, e.g., [22,23] and references therein). The most potent derivative, **2**, was chosen to be formulated with cholesteryl-poly(allylamine) (**Ch5**) polymer [24]. The hydrodynamic size of **Ch5** (1 mg/mL) formed in aqueous solution was 189 nm (see Table 2). The low polydispersity index indicated that fairly uniform single sized populations were produced. The polydispersity index is calculated between 0 and 1, the closer the value is to 0, the more monodispersity is reached. It is desirable to have such a value with <0.3 for pharmaceutical products. Interestingly, core expansion was observed when the nano-aggregates were loaded with **2** and the size increased to 286 nm but the polydispersity index only marginally increased. Additionally, the surface charge of the aggregates formed did not change after drug loading (**Ch5**+**2**) compared with the unloaded aggregate (**Ch5**) as reflected in the Zeta potential (Table 2).

**Figure 4:**
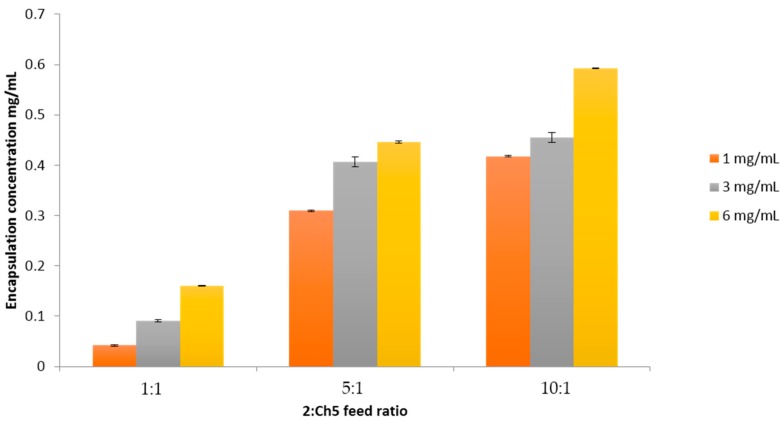
Drug encapsulation at three different feed ratios, 1:1, 5:1, and 10:1, between **2** and the polymer **Ch5**. Three initial concentrations of **2** were used: 1, 3, and 6 mg/mL (*n* = 3, ±SD).

Loading studies showed that at increased polymer concentrations, an enhanced incorporation of **2** into the hydrophobic core was achieved. Furthermore, increasing the feed ratio between compound **2** and the polymer resulted in enhanced water solubility, as shown in Figure 4. The maximum compound solubilisation observed was 6 mg/mL at a 10:1 ratio, with 0.59 mg/mL drug encapsulation being achieved.

**Figure 5:**
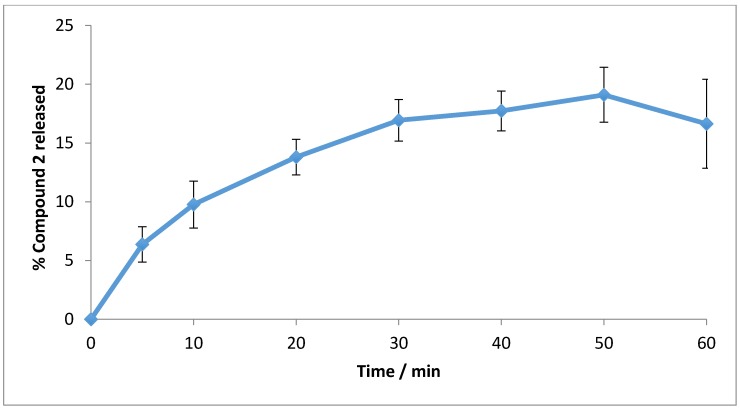
Compound **2** release from the polymer matrix into PBS at 37 °C for 1 h (*n* = 3, ±SD).

Release studies showed steady leaching of **2** from the polymer ensemble into the aqueous phase, plateauing after ~40 min as shown in Figure 5.

**Figure 6:**
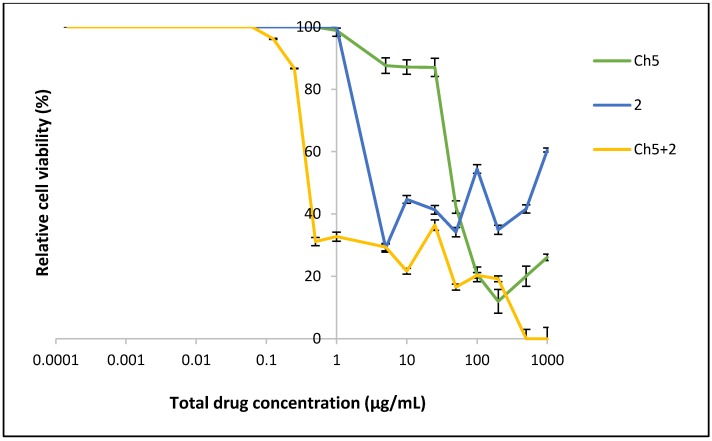
The viability of BxPC-3 cells after exposure to the polymer (**Ch5**), **2** (drug) and encapsulated drug (**Ch5**+**2**) for 24 h (*n* = 6, ±SD).

Finally the viability was measured with the MTT assay [25] in human pancreatic adenocarcinoma (BxPC-3) cells using **2** with and without the polymer encapsulation; the results are shown in Figure 6.

As can be seen in Figure 6 the cancer cell viability for the free compound **2** is effectively suppressed down to 5 μg/mL (12.95 μM) concentration with ~30% activity remaining. However, at the 1 μg/mL (2.59 μM) concentration the cells are growing without any restrictions. Thus, the IC_50_ value for free **2** can be estimated at 2.5 μg/mL (6.48 μM). Interestingly, when **2** is encapsulated into the **Ch5**-polymer there is a notable reduction in cell viability compared to the free compound. At higher concentration a complete cytotoxic effect is seen, which gradually grows to 30–35% viability as the concentration is lowered and reduces further to zero inhibition at concentrations lower than 0.29 μg/mL (0.75 μM). The IC_50_ value of **Ch5**+**2** was observed at 0.5 μg/mL (1.30 μM).

**Figure 7:**
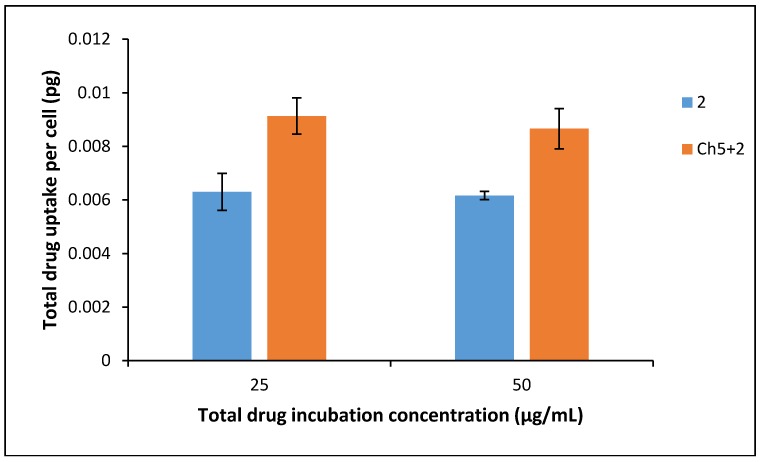
Drug uptake into BxPC-3 cells after 4 h exposure of 25 µg/mL and 50 µg/mL drug and formulation (*n* = 3, ±SD).

These results show that encapsulating **2** in the polymer matrix increases its efficacy five-fold (×5), thereby vindicating this approach for the thieno[2,3-*b*]pyridines. The most direct explanation for the enhanced efficacy is simply the increased presence of **2** in the aqueous phase, leading to more material reaching the cancer cells. Ideally, the polymer should be non-cytotoxic however, as **Ch5** has an IC_50_ of ~50 μg/mL a synergistic effect between **2** and **Ch5** is possible. An effective viability suppression is seen for the **Ch5**+**2** complex at 0.1 μg/mL, considering the mild cytotoxic effect of the **Ch5** polymer, it can be argued that it is unlikely that the polymer contributes to the anticancer efficacy. Finally, it is known that after encapsulation into nanoparticles, drug molecules are capable of more rapid cellular internalisation due to endocytosis when compared to free drugs [26]. Free drugs often enter through the cellular tight junctions or other transport systems, which can be more time-consuming than an active transport process [27]. This phenomenon can be observed in the drug uptake studies, whereby increased intracellular drug concentrations were apparent from cells incubated with the **Ch5**+**2** compared with the free drug as demonstrated in Figure 7. This trend was consistent at both concentrations tested.

## 3. Materials and Methods

### 3.1. Drug Solubility

Intrinsic aqueous drug solubility was determined by forming a supersaturated solution of the drug compound in water. The suspension was sonicated for 15 min and centrifuged at 13,000 rpm for 6 min. After 15 min the supernatant was decanted and the total drug content solubilised was analysed using reverse phase HPLC. The solution was analysed using an Agilent 1260 Infinity/DAD (100–900 nm) (Waldbronn, Germany) with a Zorbax eclipse XDB (8.5 μm, 4.6 mm × 150 mm) (Waldbronn, Germany) column. The mobile phase comprised of 80:20 acetonitrile: H_2_O containing 45 mM ammonium formate buffered to pH 3.5 with formic acid. Flow rate was 1 mL/min with 5 μL injection volume.

### 3.2. Cell Proliferation Assay

As described in detail previously [28] cell proliferation was measured using a thymidine incorporation assay by seeding 3000 cells in each well using 96-well plates with varying concentrations of inhibitors for three days. Experiments were performed in triplicate with a minimum of two experimental repeats. Briefly, 0.04 µCi of ^3^H-thymidine was added to each well and incubated for five hours, after which the cells were gathered onto glass fibre filters using an automated TomTec harvester. Filters were incubated with Betaplate Scint and thymidine incorporation determined with Trilux/Betaplate counter showing the percentage of cells incorporated with ^3^H-thymidine into the DNA helix.

Furthermore, derivatives **3**, **4**, and **6** were sent to the National Cancer Institute’s Developmental Therapeutic Program (DTP) where they were screened against a panel of sixty human tumour cell lines (NCI60, for further information see [18,29,30] and a complete description in the Supplementary Materials section).

### 3.3. Preparation of Cholesteryl-Poly(allylamine) (***Ch5***)

**Figure 8:**
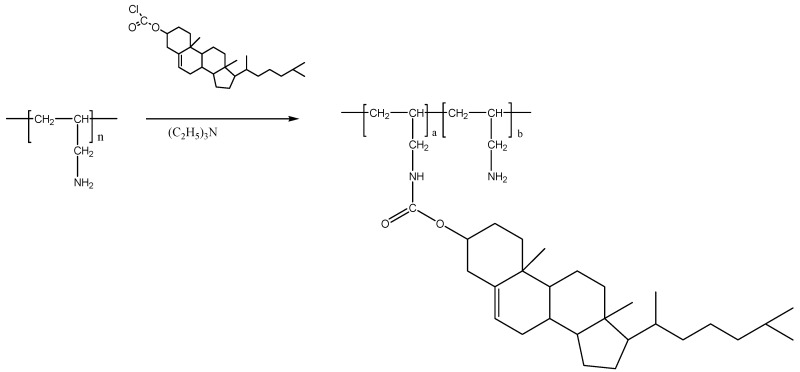
Chemical synthesis of cholesterol-PAA (**Ch5**).

Cholesteryl chloride was grafted in 5% molar ratio to PAA as shown in Figure 8.

#### 3.3.1. ^1^H NMR Spectra of **Ch5**

The **Ch5** spectrum (see Figure S1) had peak assignments at: δ_0.75_, δ_1.10_, δ_1.15_, and δ_1.25_ = CH_3_ (cholesteryl), δ_1.4–2.0_ = CH_2_ (cholesteryl, PAA), δ_2.60_ = CH_2_ (cholesteryl), δ_2.75–3.00_= CH_2_ (attached to –NH and –NH_2_ groups on PAA), δ_4.5_ = CH=O (carbonyl bond), δ_5.5_ = C=CH (cholesteryl). The peaks at δ_3.25_ and δ_4.75_ were due to water and MEOD respectively. The degree of hydrophobic modification could be calculated by comparing the peak at δ_0.75_ (cholesteryl) with the peaks at δ_2.75–3.20_ (–CH_2_ attached to –NH and –NH_2_ groups) on PAA backbone as follows: (Integration at δ_0.75_/3)/(Integration at δ_2.75–3.00_/2) × 100% = 3.6% hydrophobic substitution.

#### 3.3.2. Elemental Analysis of **Ch5** Polymer

Elemental analysis. Modified polymer samples (1 mg) were analysed for abundance of carbon, hydrogen, and nitrogen (and halogen content) using a Perkin Elmer series 2 elemental analyser (Perkin Elmer, Oswestry, UK).

The elemental analysis results indicated that the actual % mole hydrophobic grafting per PAA monomer with **Ch5** were in good agreement with the initial molar feed ratio (Table S8).

#### 3.3.3. FTIR of **Ch5** Polymer

FTIR analysis of all the polymer was carried out with a Perkin Elmer (Spectrum BX, Oswestry, UK), fitted with a diamond powder tip. The polymers (5 mg) were placed under the diamond tip and 64 scans were run following a background correction. The FTIR spectrum for **Ch5** (Figure S2) shows the presence of absorbance for the bonds present within the polymer (Table S9). The peak at 2357 cm^−1^ is due to C–O arising from background CO_2_ and the broad peak at 3293 cm^−1^ is due to O–H of water, which has been absorbed by the hygroscopic polymer. The presence of C=O and C–O peaks due to absorbance at 1464 cm^−1^ and 1141 cm^−1^, respectively, indicating the presence of the carbonyl group between the backbone and the cholesteryl moiety. 

Photon correlation spectroscopy: Polymeric self-assemblies were formed by probe sonication of the polymers (1 mg/mL) in doubly distilled water before filtering with 0.45 µm syringe filter. Hydrodynamic diameters and polydispersity index measurements were carried out using a photon correlation spectrometer (Zetasizer Nano-ZS, Malvern Instruments, Malvern, UK). All measurements were conducted in triplicate at 25 °C and an average value was determined.

### 3.4. Drug Loading

Polymer solutions of 1 mg/mL, 3 mg/mL, and 6 mg/mL were made up in deionised water and probe sonicated for 10 min to ensure aggregation of the self-assemblies was achieved. Compound **2** was added in 1:1, 5:1, and 10:1 initial drug: excipient weight ratios and the drug-polymer solutions were probe sonicated for a further 10 min to ensure maximum encapsulation was achieved. After cooling to room temperature, the solutions were filtered using 0.45 µm syringe filters (with prefilters) to ensure any excess un-encapsulated drugs were removed. Drug quantification was carried out using UV-VIS spectroscopy with the samples diluted in DMSO. Absorbance was measured at 304 nm. Calibration was run from 0.0039–0.0313 mg/mL with R^2^ = 0.992 (see Supplementary Materials Figure S3 for the calibration plot).

### 3.5. In Vitro Drug Release Studies 

The formulation (1 mL) was pipetted inside dialysis tubing (MW cut off = 12–14 kDa) and dialysed against PBS in sink (100 mL, 0.2 M) at 37 °C with stirring. At various time points 1 mL of the external PBS was extracted and replaced with 1 mL of fresh PBS. The presence of drug in the collected PBS was determined using UV-VIS spectroscopy as before.

### 3.6. Cytotoxicity of Formulation Using MTT Assay 

An MTT assay was carried out using human pancreatic adenocarcinoma (BxPC-3) cells. Cells were cultured in RPMI medium containing 10% FBS and 1% penicillin streptomycin (Penstrep). Cytotoxicity assay for compound **2** alone (1–1 × 10^−4^ mg/mL) and formulations of the **Ch5**+**2** polymer was carried out. The experiments were performed in triplicate (*n* = 3) using eight wells per plate for each data point. The polymers were fixed at a concentration of 0.005 mg/mL where the cell viability is 90% (IC_90_) based on the MTT assay. Polymeric self-assemblies were formed by probe sonicating the polymers in sterile water (5 mg/mL). The polymer stock solutions were diluted to 0.005 mg/mL with media. A drug stock solution (20 mg/mL) was prepared by diluting compound **2** in DMSO. The formulations were prepared by addition of compound **2** into 0.005 mg/mL polymer solution.

### 3.7. Drug Uptake into BxPC-3 Cells

Cells (3 mL, 150,000 cells/well) were seeded into six-well plates and incubated for 24 h at 37 °C with 5% CO_2_. The media was replaced with 25 μg/mL and 50 μg/mL of either **2** or **Ch5+2** and incubated for 4 h. The medium was removed and each well was washed with 1 mL PBS before the addition of 185 μL trypsin into each well. Cells re-suspended in 1 mL media and viable cells were counted using an automated cell counter (Invitrogen Countess^®^, Loughborough, UK). Cells (100,000) were transferred into Eppendorf tubes and centrifuged (800 rpm, 5 min). The supernatant was removed and cells were resuspended in DMSO and was quantified as previously described.

### 3.8. Molecular Modelling

The compounds were docked to the crystal structure of PLC-δ1 (PDB ID: 1DJX, resolution 2.3 Å) [31], TDP1 (PDB ID: 1MU7, resolution 2.0 Å) [32], Atox1(PDB ID:1FEE, resolution 1.8 Å) [33], tubulin-colchicine complex (PDB ID: 4O2B, resolution 2.3 Å) [34], and A_2A_AR (PDB ID: 3EML, resolution 2.6 Å) [35], which was obtained from the Protein Data Bank (PDB) [36,37] using the GoldScore (GS) [38], ChemScore (CS) [39,40], ChemPLP [41], and ASP [42] scoring functions were implemented to validate the predicted binding modes and relative energies of the ligands using the GOLD v5.2 software suite. Fifty docking runs were allowed for each ligand with default search efficiency (100%) with 10 Å radiuses. The basic amino acid residues lysine and arginine were defined as protonated. Furthermore, aspartic and glutamic acids were assumed to be deprotonated. The Scigress Ultra version F.J 2.6 program [43] was used to prepare the crystal structures for docking, i.e., hydrogen atoms were added, the co-crystallised heavy ions and ligands as well as crystallographic water molecules were removed. The Scigress software suite was also used to build the inhibitors and the MM2 [44] force field was used to optimise the structures. For PLC-δ1, the centre of the binding pocket was defined as the position of the Ca^2+^ ion (*x* = 78.799, *y* = 38.878, *z* = −19.069), for tubulin the colchicine pocket is used as the binding centre (*x* = 13.222, *y* = 8.371, *z* = −23.331), whilst A_2A_AR obtained good consensus upon re-docking of the co-crystalized ligand using (*x* = −9.42, *y* = −9.544, *z* = 56.644) coordinates. For TDP1, the centre of the binding pocket was defined as the position of the tungsten (W) of the co-crystallised ligand (*x* = 8.312, *y* = 12.660, *z* = 34.452), while, for Atox1, the centre of the binding pockets were defined as the position of the sulphur of Cys12 (*x* = 66.121, *y* = −55.634, *z* = 36.866).

The QikProp 3.2 [45] software package was used to calculate the molecular descriptors of the compounds. The reliability of the prediction power of QikProp is established for the molecular descriptors used in this study [46].

## 4. Conclusions

In this work, two strategies were explored in order to enhance the water solubility of the thieno[2,3-*b*]pyridine anticancer compounds. First, a morpholine moiety was attached to the molecular scaffold by substituting the sulphur atom for a nitrogen, leading to the 1*H*-pyrrolo[2,3-*b*]pyridine molecular framework. An excellent increase in solubility was achieved but, concurrently, the anticancer efficacy was severely diminished. Molecular modelling against the putative targets of the thieno[2,3-*b*]pyridines did not give a conclusive result. The lack of efficacy can be hypothesised by poor cell permeability of the 1*H*-pyrrolo[2,3-*b*]pyridine ligands. Alternatively, an unknown molecular target could be responsible for the efficacy of the thieno[2,3-*b*]pyridines. Second, as an alternative strategy, thieno[2,3-*b*]pyridine derivative **2** was loaded into the cholesteryl-poly(allylamine) polymer, forming nanoparticles. The complex was tested against the BxPC-3 pancreatic cancer cell lines and showed excellent potency with a measured IC_50_ value of 0.5 μg/mL (1.30 μM) as compared with 2.5 μg/mL (6.48 μM), which is five times more potent than the free derivative, **2**.

## Figures and Tables

**Table 1:** The experimental and calculated aqueous solubility(S) of derivatives **1**–**7**, as well as their predicted log P.

	Experimental S			Calculated S	
	μg/mL	log S ^a^	μg/mL	log S ^a^	log P
**1**	1.21	−5.4	13.4	−4.4	2.9
**2**	3.53	−5.0	96.9	−3.6	2.2
**3**	1339.8/1336.7 ^b^	−2.5 ^c^	1089.1	−2.6	2.9
**4**	0.20	−6.3	1.1	−5.6	5.3
**5**	0.18	−6.3	5.9	−4.8	4.0
**6**	0.76	−5.7	27.7	−4.1	3.3
**7**	0.61	−5.8	13.9	−4.4	3.5

^a^ Units are in mol/L. ^b^ Two measurements made. ^c^ Avarage of the two measurements.

**Table 2:** The hydrodynamic diameter with the corresponding polydispersity index and the zeta potential measurement of the aggregates in water (*n* = 3, ±SD).

	Hydrodynamic Diameter (nm)	Polydispersity Index	Zeta Potential, mV
**Ch5**	189 ± 1.800	0.246 ± 0.010	+39.6 (2.242)
**Ch5+2**	286 ± 5.839	0.267 ± 0.011	+36.5 (1.635)

## Supplementary Materials

Supplementary materials are available online.
