# Supplementary material for: Investigation into Improving the Aqueous Solubility of the Thieno[2,3-b]pyridine Anti-Proliferative Agents
Source: Molecules. 2018 Jan 11;23(1):145. doi: 10.3390/molecules23010145 (PMC6017400; doi:10.3390/molecules23010145)

Supplementary Information

Investigation into improving the aqueous solubility of the thieno[2,3-*b*]pyridine anti-proliferative agents

Ayesha Zafar[1], Lisa I. Pilkington[1], Natalie A. Haverkate[1], Michelle van Rensburg[1], Euphemia Leung[2],Sisira Kumara[2], William A. Denny[2], David Barker[1], Ali Alsuraifi[3], Clare Hoskins[3] and Jóhannes Reynisson[1]*

[1]School of Chemical Sciences, University of Auckland, New Zealand

[2]Auckland Cancer Society Research Centre and Department of Molecular Medicine and Pathology, University of Auckland, New Zealand

[3]Institute for Science and Technology in Medicine, Keele University, Guy Hilton Research Centre, Stoke-on-Trent, United Kingdom

Index

Table S1. Calculated molecular descriptors for the derivatives. Page 2

Table S2. The results of the thymidine uptake and SRB assays. Page 2

Table S3. Interactions and scores for PLC-δ1. Page 2

Table S4. Interactions and scores for TDP1. Page 3

Table S5. Interactions and scores for Atox1. Page 3

Table S6. Interactions and scores for A_{2A}AR. Page 4

Table S7. Interactions and scores for Tubulin. Page 4

Figure S1. ^{1}H NMR spectra of **Ch5** polymer. Page 5

Table S8. Elemental analysis of **Ch5** polymer. Page 5

Figure S2. FTIR of freeze dried **Ch5** polymer. Page 6

Table S8. Peak bandwidth assignment occurring on FTIR spectrum. Page 7

Figure S3. Compound **2** Uv/vis calibration. Page 8

Protocol. NCI's 60-cell line panel growth inhibition assay**.** Page 8

NCI data Pages 9-11

Table S1. Calculated molecular descriptors for the derivatives.

Molecule	MW	log P	DonorHB	AccptHB	PSA	RB	log S
**1**	337.4	2.2	2	5.5	99.4	3	-3.6
**2**	385.9	2.9	2	5.5	97.7	3	-4.5
**3**	433.6	2.9	2	7.2	76.0	7	-2.6
**4**	424.5	5.3	2	3.5	63.0	7	-5.6
**5**	370.4	4.0	2	3.5	63.5	3	-4.8
**6**	348.4	3.3	2	3.5	65.2	3	-4.1
**7**	348.4	3.5	2	3.5	68.4	3	-4.4

Table S2. The results of the thymidine assays at 1 μM concentration. The average relative growth is given in percentages (%) as compared to untreated cells at 100% growth, i.e., the lower percentage numbers represent greater growth inhibition.

	**MDA-MB-231**	**HCT116**
**3**	99.5	90.3
**4**	105.4	103.6
**5**	97.2	102.1
**6**	106.0	104.2
**7**	100.7	99.0

Table S3. Predicted interactions and scores for the thienopyridines with PLC-δ1.

**Molecules**	**Hydrogen Bonding residues**	**GS**	**CS**	**ASP**	**PLP**
**1**	His356, Arg549,<br>Glu341, Lys438	53.9	30.2	34.6	61.5
**2**	His311, Arg549, Asn312. Lys438, Glu341	57.4	31.2	34.2	63.6
**3**	His356, Asn312,<br>Glu341	63.9	28.1	43.5	74.9
**4**	His311, Asn312,<br>Glu341	63.9	30.2	44.5	83.9
**5**	Glu390	53.5	28.5	35.3	62.3
**6**	His356, Asn312, Glu341	51.0	28.5	34.1	59.9
**7**	His356, Asn312, Glu341	59.0	26.8	32.1	63.0

Table S4. Predicted interactions and scores for the thienopyridines with TDP1.

Molecules	Hydrogen Bonding residues	GS	CS	ASP	PLP
**1**	Ser400, His493	52.6	29.2	30.1	48.6
**2**	Asn516, His493, Asn283	49.9	28.2	32.5	47.6
**3**	Tyr204, Ser518	52.8	26.6	39.4	58.7
**4**	No H-bonding	52.5	28.7	39.2	61.4
**5**	His493	50.6	28.9	34.4	53.1
**6**	His263, His493, Asn516	51.5	29.5	33.0	53.1
**7**	His263, His493	54.2	27.7	33.0	49.5

Table S5. Predicted interactions and scores for the thienopyridines with Atox1.

Molecules	Bonding residues	GS	CS	ASP	PLP
**1**	Thr58, π-π stacking with Lys60,Cys15	40.3	20.3	14.9	39.1
**2**	Arg21,π-π stacking with Lys60	44.4	20.8	15.1	39.9
**3**	Lys60,Interaction with Thr58	40.0	19.6	19.6	50.7
**4**	Thr58, π-π stacking with Lys60,Cys15	40.6	21.1	21.8	56.5
**5**	Gly31	36.7	23.2	18.8	50.8
**6**	π-π stacking with Lys60	41.3	19.5	15.0	43.2
**7**	Thr58, Gly14, π-π stacking with Lys60	40.2	19.8	14.7	42.2

Table S6. Predicted interactions and scores for the thienopyridines with $A_{2A}AR$.

Molecules	Hydrogen Bonding residues	GS	CS	ASP	PLP
**1**	Asn253,Glu169, stacking interaction with Phe168	61.0	36.7	39.8	67.4
**2**	Asn253,Glu169, stacking interaction with Phe168	67.2	44.1	42.2	69.0
**3**	Asn253, stacking interaction with Phe168	74.9	42.0	44.5	92.5
**4**	Asn253,Glu169, stacking interaction with Phe168	71.9	45.9	49.7	96.2
**5**	Asn253, Glu169, stacking interaction with Phe168	60.6	43.6	45.5	77.0
**6**	Asn253, stacking interaction with Phe168	62.5	41.5	41.6	73.1
**7**	Asn253, Glu169, stacking interaction with Phe168	64.8	41.2	41.9	74.1

Table S7. Predicted interactions and scores for the thienopyridines with the Tubulin-colchicine site.

Molecules	Hydrogen Bonding residues	GS	CS	ASP	PLP
**1**	Buried inside pocket like colchicine	62.5	29.6	28.1	61.3
**2**	Buried inside pocket like colchicine	63.9	31.4	25.4	54.9
**3**	Thr179	72.7	33.9	30.6	97.1
**4**	Thr179	79.1	36.4	36.3	99.8
**5**	Buried inside pocket like colchicine	56.7	33.7	28.5	61.5
**6**	Thr179	61.3	29.5	22.9	67.2
**7**	Thr179	51.8	30.0	21.5	70.6

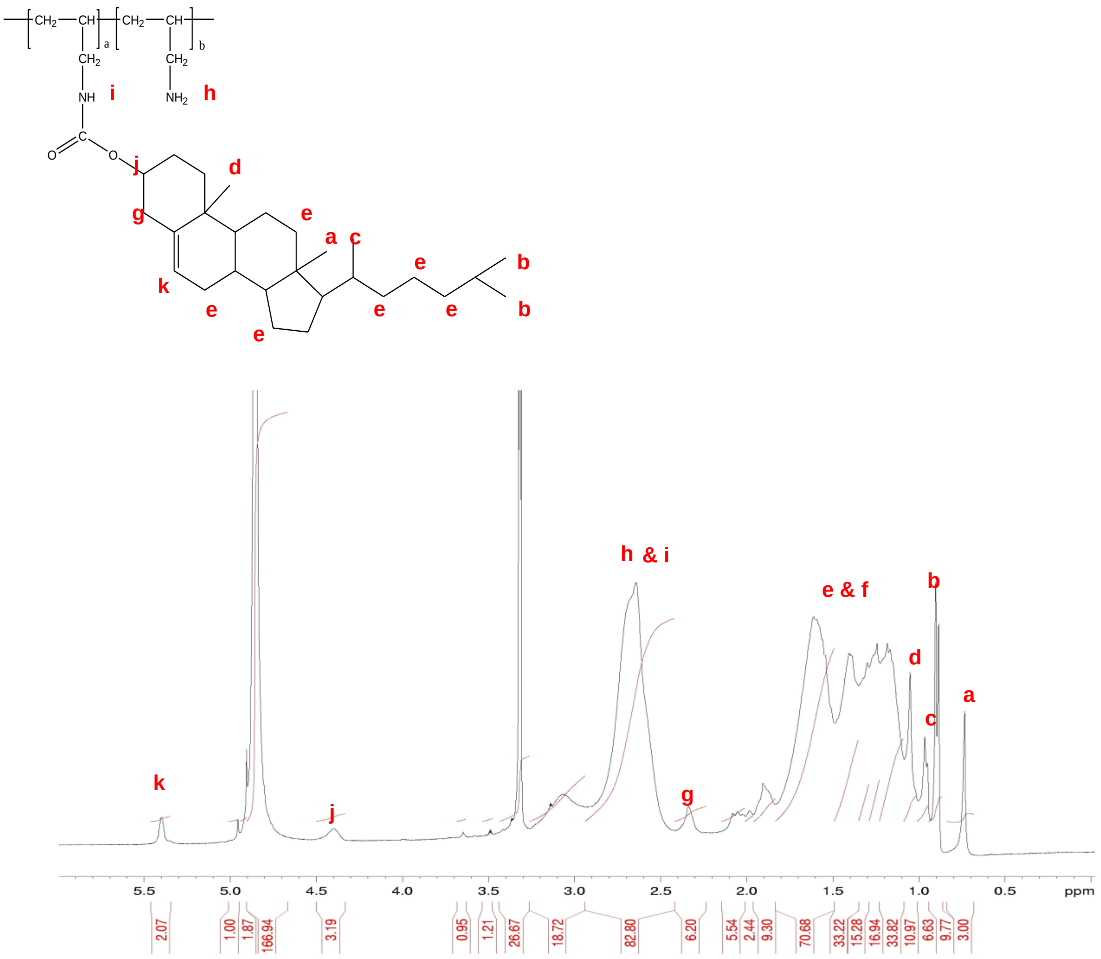

Figure S1.^{1}H NMR spectra of **Ch5** polymer in MeOD carried out using 400MHz NMR at 25 °C.

Table S8. Elemental analysis of **Ch5** polymer.

Polymer	Initial monomer:hydrophobic pendant group molar feed ratio	% Mole hydrophobic grafting per PAA monomer (n=3,±SD)	% Yield (n=3,±SD)
Ch5	1:0.005	4.6 (1.2)	79.5 (10.2)

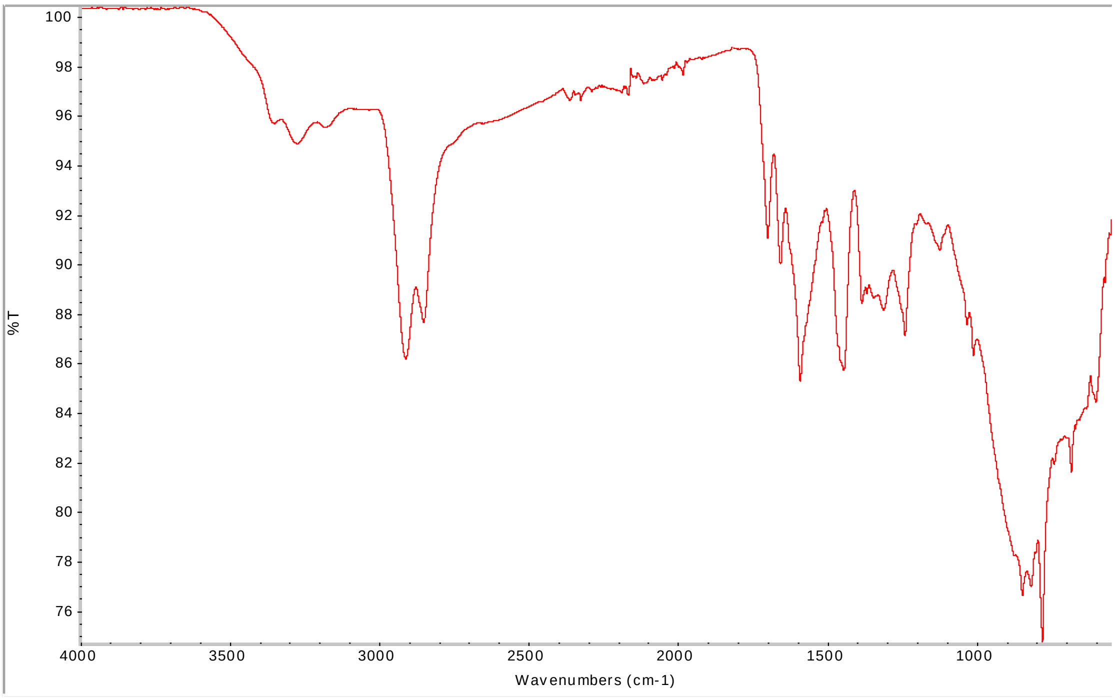

Figure S2. FTIR of freeze dried **Ch5** polymer.

Table S8. Peak bandwidth assignment occurring on FTIR spectrum of **Ch5** using diamond powder tip (64 scans).

Polymer / Formulation	*Bandwidth (cm^{-1})*	*Bond type*	*Functional Group*
PAA	3361<br>1595	N-H Stretch	1 ˚Amine
	2913<br>2854<br>1373<br>1316	C-H Stretch	Alkyl
	1450	C-C Bend	Alkyl
	925<br>909	C-N Bend	
**Ch5**	1450	C-C Bend	Alkyl
	925<br>909	C-N Bend	
	1464<br>815	C=C Bend	Aromatic
	1457	C-C Bend	Alkyl
	1383<br>1312	C-H Bend	Alkyl
	1141<br>930	C-O Bend	Carbonyl

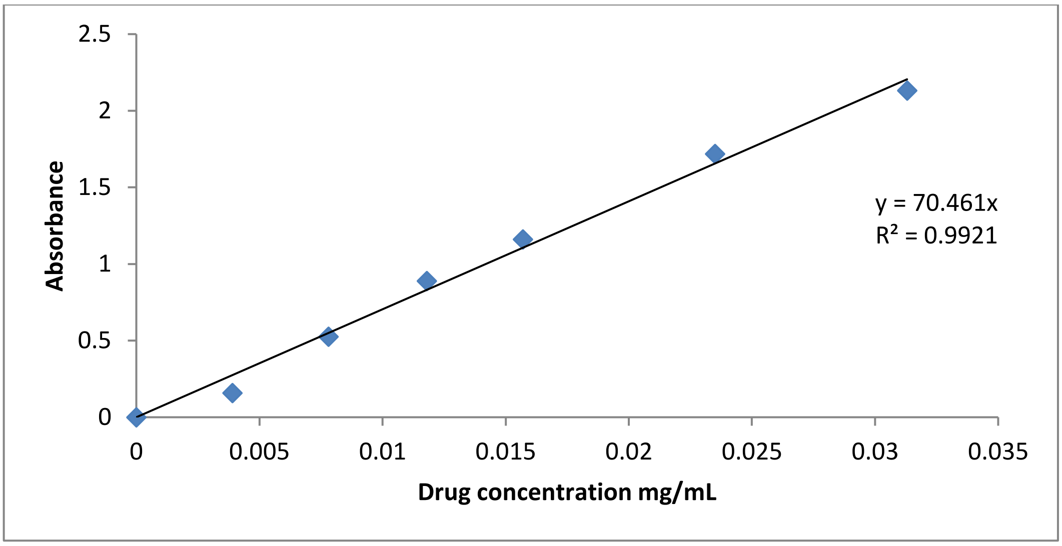

Figure S3. Compound **2** UV-vis calibration in DMSO at 304 nm.

NCI's 60-cell line panel growth inhibition assay

The NCI's human 60-cell lines were grown in RPMI 1640 medium containing 5% FBS and 2mM L-glutamine. Cells were inoculated into 96-well plates at plating densities 5000–40 000 cells per well, based on the doubling time of individual cell lines. Plates were then incubated at 37 °C, 5% CO_2, 95% air and 100% relative humidity for 24 h prior to addition of tested compounds. After 24 h, two plates of each cell line were fixed *in situ* with trichloroacetic acid (TCA), to represent a measurement of the cell population for each cell line at the time of tested compound addition. Tested compounds were solubilized in DMSO at a concentration 400 times that of the desired final maximum test concentration and stored frozen prior to use. An aliquot of each frozen tested concentrate was thawed and diluted to twice the desired final maximum test concentration with complete medium containing 50 µg mL^{-1} gentamicin. 100 µL aliquot of the tested drug diluted solution was added to appropriate wells containing 100 µL of medium, resulting in the required final drug doses. Following tested compound addition, plates were incubated for additional 48 h. The assay was terminated by the addition of cold TCA for adherent cells. Cells were fixed *in situ* by addition of 50 µL of cold 50% (w/v) TCA (final concentration, 10% TCA) and incubated for 60 min at 4 °C. The supernatant was discarded, and plates were washed 5 times with water and air dried. Sulforhodamine B (SRB) solution (100 µL), 0.4%(w/v) in 1% acetic acid was added to each well, and plates were incubated for 10 min at rt. After staining, the unbound dye was removed by washing five times with 1% acetic acid and plates were air dried. The bound stain was subsequently solubilized with 10 mM Trizma base, and the absorbance was measured on a plate reader at 515 nm. For suspension cells, the methodology was identical except the assay termination by fixing settled cells at the bottom of each well by adding 50 µL of 80%TCA (final concentration, 16% TCA).
Taken from: K. A. El Sayed, A. I. Foudah, A. M. S. Mayer, A. M. Crider and D. Song, *Med. Chem. Comm.*, 2013, **4**, 1231-1238.

NCI Data

Derivative 3

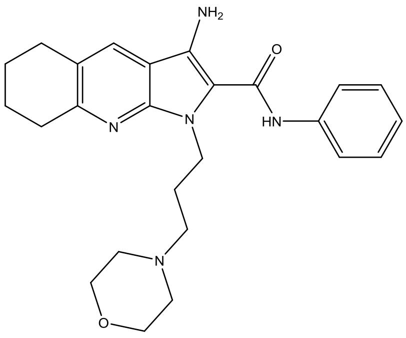

Developmental Therapeutics Program

One Dose Mean Graph

NSC: D-793686 / 1 | **Conc:** 8.00E-6 Molar | **Test Date:** Oct 24, 2016

Experiment ID: 1610OS04 | **Report Date:** May 24, 2017

Panel/Cell Line	Growth Percent
**Leukemia**	
CCRF-CEM	100.44
HL-60(TB)	98.57
K-562	96.34
MOLT-4	91.80
RPMI-8226	99.96
SR	93.28
**Non-Small Cell Lung Cancer**	
A549/ATCC	99.73
EKVX	99.15
HOP-62	87.39
HOP-92	96.35
NCI-H226	91.40
NCI-H23	96.45
NCI-H322M	101.31
NCI-H460	104.43
NCI-H522	89.28
**Colon Cancer**	
COLO 205	105.26
HCC-2998	108.69
HCT-116	103.83
HCT-15	111.12
HT29	103.68
KM12	100.69
SW-620	96.36
**CNS Cancer**	
SF-268	94.38
SF-295	100.19
SF-539	96.54
SNB-19	102.90
SNB-75	82.50
U251	102.55
**Melanoma**	
LOX IMVI	93.75
MALME-3M	98.19
M14	99.61
MDA-MB-435	107.59
SK-MEL-2	96.96
SK-MEL-28	107.91
SK-MEL-5	101.88
UACC-257	104.84
UACC-62	96.88
**Ovarian Cancer**	
IGROV1	107.32
OVCAR-3	103.46
OVCAR-4	112.10
OVCAR-5	106.17
OVCAR-8	102.74
NCI/ADR-RES	98.23
SK-OV-3	98.37
**Renal Cancer**	
786-0	105.53
A498	83.19
RXF 393	100.80
SN12C	100.68
TK-10	103.34
UO-31	79.08
**Prostate Cancer**	
PC-3	91.59
DU-145	108.27
**Breast Cancer**	
MCF7	91.09
MDA-MB-231/ATCC	99.18
HS 578T	94.97
BT-549	99.36
T-47D	91.62
MDA-MB-468	109.03
Mean	99.11
Delta	20.03
Range	33.02

Mean Growth Percent - Growth Percent

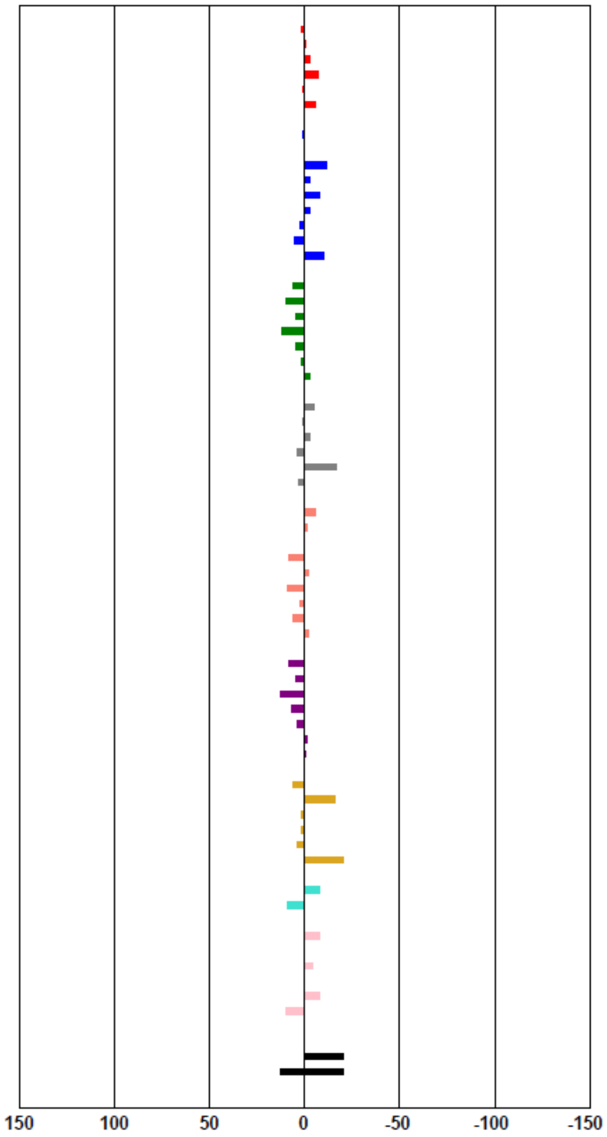

Derivative 4

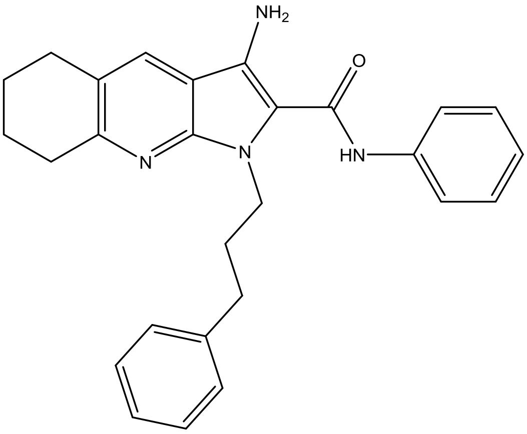

Developmental Therapeutics Program	NSC: D-793687 / 1	Conc: 1.00E-5 Molar	Test Date: Oct 24, 2016
One Dose Mean Graph	Experiment ID: 1610OS04		Report Date: May 24, 2017

Panel/Cell Line	Growth Percent
Leukemia	
CCRF-CEM	95.06
HL-60(TB)	87.09
K-562	85.51
MOLT-4	72.96
RPMI-8226	80.48
SR	87.38
Non-Small Cell Lung Cancer	
A549/ATCC	98.23
EKVX	80.58
HOP-62	88.76
HOP-92	83.15
NCI-H226	77.31
NCI-H23	78.22
NCI-H322M	92.49
NCI-H460	98.27
NCI-H522	68.38
Colon Cancer	
COLO 205	101.54
HCC-2998	111.56
HCT-116	79.78
HCT-15	94.01
HT29	98.56
KM12	95.23
SW-620	89.99
CNS Cancer	
SF-268	92.62
SF-295	98.57
SF-539	99.83
SNB-19	99.11
SNB-75	79.63
U251	93.18
Melanoma	
LOX IMVI	89.04
MALME-3M	105.56
M14	99.75
MDA-MB-435	109.79
SK-MEL-2	86.19
SK-MEL-28	111.00
SK-MEL-5	87.96
UACC-257	109.00
UACC-62	77.63
Ovarian Cancer	
IGROV1	86.45
OVCAR-3	94.27
OVCAR-4	83.35
OVCAR-5	104.58
OVCAR-8	98.04
NCI/ADR-RES	91.11
SK-OV-3	96.36
Renal Cancer	
786-0	99.38
A498	80.90
RXF 393	95.20
SN12C	94.53
TK-10	115.24
UO-31	68.28
Prostate Cancer	
PC-3	86.88
DU-145	104.46
Breast Cancer	
MCF7	78.95
MDA-MB-231/ATCC	87.75
HS 578T	90.69
BT-549	101.34
T-47D	70.88
MDA-MB-468	105.45
Mean	91.68
Delta	23.40
Range	46.96

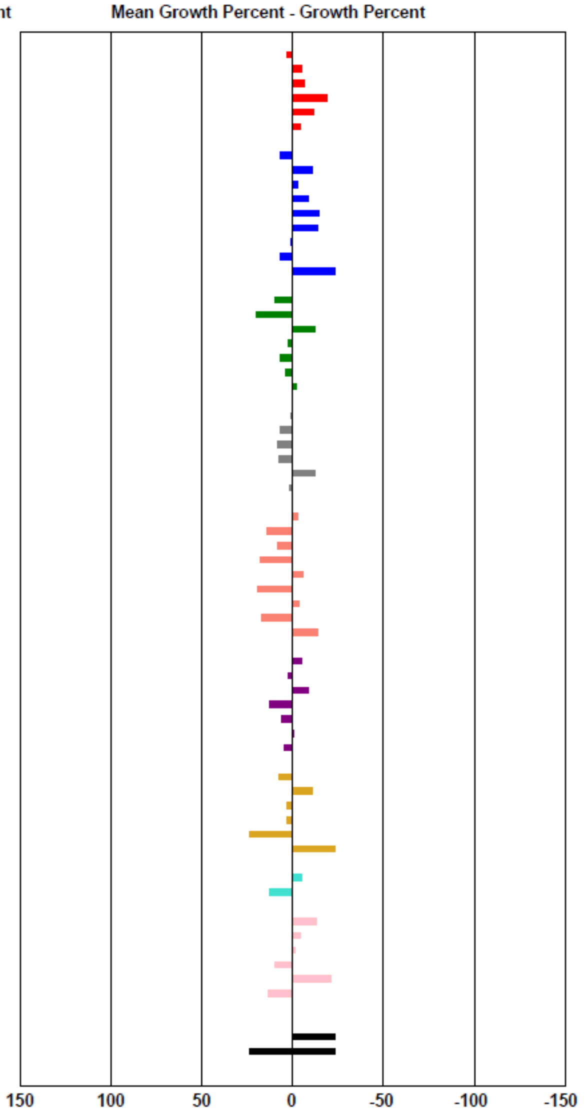

Derivative 6

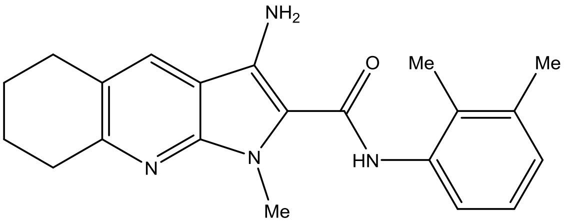

Developmental Therapeutics Program One Dose Mean Graph	NSC: D-793689 / 1	Conc: 1.00E-5 Molar	Test Date: Oct 24, 2016
	Experiment ID: 1610OS04		Report Date: May 24, 2017

Panel/Cell Line	Growth Percent
**Leukemia**	
CCRF-CEM	72.89
HL-60(TB)	67.03
K-562	45.54
MOLT-4	81.06
RPMI-8226	81.08
SR	47.66
**Non-Small Cell Lung Cancer**	
A549/ATCC	72.08
EKVX	79.76
HOP-62	71.71
HOP-92	58.13
NCI-H226	83.65
NCI-H23	85.05
NCI-H322M	78.86
NCI-H460	57.09
NCI-H522	60.56
**Colon Cancer**	
COLO 205	87.50
HCC-2998	94.41
HCT-116	58.92
HCT-15	70.50
HT29	78.85
KM12	85.57
SW-620	74.77
**CNS Cancer**	
SF-268	89.43
SF-295	71.44
SF-539	84.75
SNB-19	81.92
SNB-75	65.48
U251	79.22
**Melanoma**	
LOX IMVI	80.91
MALME-3M	75.87
M14	81.69
MDA-MB-435	53.92
SK-MEL-2	80.84
SK-MEL-28	91.25
SK-MEL-5	83.12
UACC-257	90.34
UACC-62	77.60
**Ovarian Cancer**	
IGROV1	63.89
OVCAR-3	94.40
OVCAR-4	55.20
OVCAR-5	90.13
OVCAR-8	80.93
NCI/ADR-RES	71.50
SK-OV-3	83.76
**Renal Cancer**	
786-0	78.96
A498	76.53
RXF 393	90.47
SN12C	83.55
TK-10	92.91
UO-31	76.26
**Prostate Cancer**	
PC-3	81.92
DU-145	96.49
**Breast Cancer**	
MCF7	68.53
MDA-MB-231/ATCC	72.39
HS 578T	84.66
BT-549	87.88
T-47D	75.38
MDA-MB-468	76.97
Mean	76.95
Delta	31.41
Range	50.95

Mean Growth Percent - Growth Percent

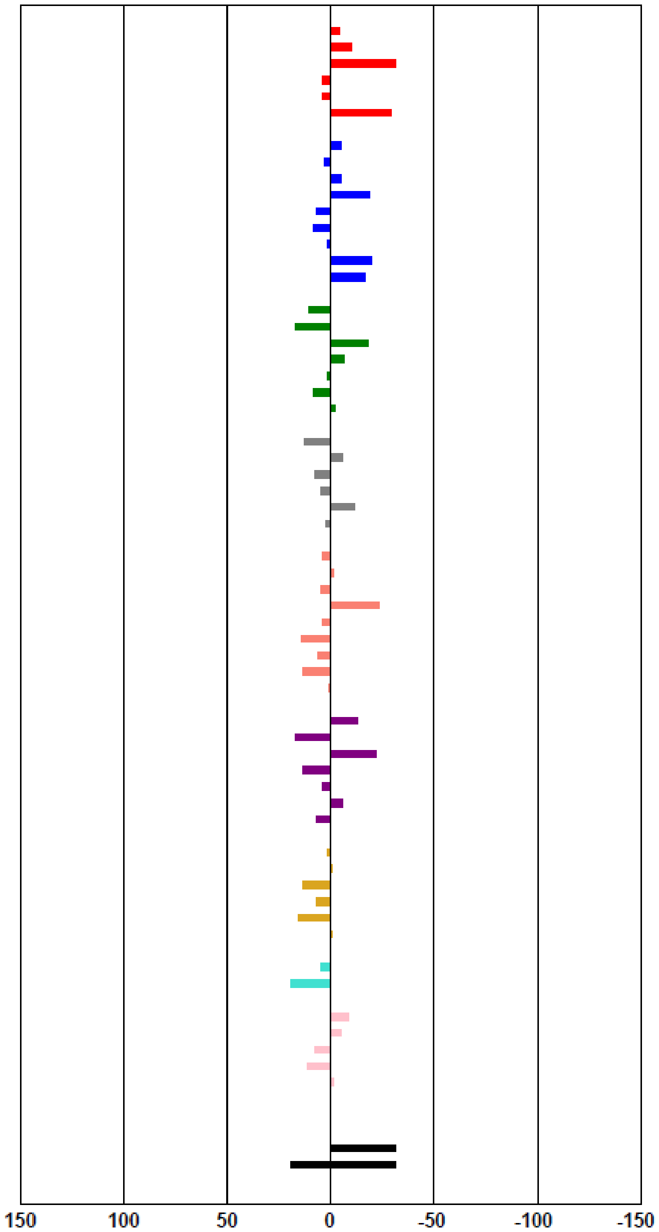

Supplement: Supplementary file 1 [file molecules-23-00145-s001.pdf]
